# G6PD Deficiency and Harilaou Variant in a Newborn: Intrauterine Haemolysis and Meconium Aspiration Syndrome

**DOI:** 10.34763/jmotherandchild.20212501.d-20-00021

**Published:** 2021-10-11

**Authors:** Kleoniki I. Athanasiadou, Maria Amarantidou, Eftychia Drogouti, Marina Economou, George Mitsiakos, Evgenia Papakonstantinou, Paraskevi Karagianni

**Affiliations:** 1 2nd Neonatal Department and Neonatal Intensive Care Unit (NICU), Aristotle University of Thessaloniki, Papageorgiou General Hospital, Thessaloniki, Greece; 2 1st Department of Pediatrics, Aristotle University of Thessaloniki, Hippokration General Hospital, Thessaloniki, Greece; 3 Pediatric Oncology Department, Hippokration Hospital, Thessaloniki, Greece

**Keywords:** G6PD deficiency, meconium aspiration syndrome, G6PD Harilaou, intrauterine haemolysis

## Abstract

G6PD deficiency is one of the most commonly inherited enzymopathies with a hallmark of an X-linked pattern. G6PD has more than 300 unique variants with different enzyme activity. The G6PD Mediterranean variant is prevalent in Greece and associated with asymptomatic patients who may experience haemolysis under specific circumstances. G6PD Harilaou is a new variant that was first described in Greece in an eight-year-old boy who suffered chronic haemolysis demanding multiple transfusions. We present a new case of the G6PD Harilaou variant in a Greek male neonate who suffered severe intrauterine haemolysis and passed away 39 hours after birth. To our knowledge, it is the second reported G6PD Harilaou case.

## Introduction

Glucose-6-phosphate dehydrogenase (G6PD) (EC 1.1.1.49) is the enzyme that catalyses the first step of the pentose phosphate pathway and produces NADPH. NADPH is required for the generation of reduced glutathione, an agent that protects red blood cells from the toxic action of reactive oxygen species (ROS). G6PD deficiency is the most commonly inherited enzymopathy and is widely spread in Greece, with a mean prevalence of 3.1% and 6.3% in Northern Greece [1,2]. G6PD deficiency demonstrates an X-linked recessive pattern of inheritance, and there are more than 300 different G6PD gene mutations identified at present [3, 4, 5]. In Greece, the G6PD Mediterranean variant [563C to T substitution] accounts for more than 77% of cases and is associated with intermittent haemolysis under specific circumstances (e.g., the use of certain medications like aspirin, sulphonamides, cotrimoxazole, nitrofurantoin, quinolone, dapsone or substances like naphthalene and fava beans). Enzyme activity varies between different G6PD mutations, and so WHO established an official explanatory classification (Table 1).

**Table 1:** WHO classification of G6PD variants according to the degree of enzyme deficiency and severity of haemolysis

**Class I**	Severe enzyme deficiency (<1% residual activity) chronic non-spherocytic haemolysis
**Class II**	Severe enzyme deficiency (1–10% residual activity) intermittent acute haemolysis
**Class III**	Moderate enzyme deficiency (10–60% residual activity) intermittent acute haemolysis
**Class IV**	No enzyme deficiency (60–150% activity)
**Class V**	Increased enzyme activity (>150%)

In 1989, Town et al. found and announced a new variant, G6PD Harilaou, named after the region of Thessaloniki, Greece, where the patient lived [6]. The G6PD Harilaou variant [738T to G substitution] was associated with chronic haemolysis and enzyme activity of less than 1% of normal. It now belongs to Class I per the WHO classification, while the G6PD Mediterranean belongs to Class II. The clinical heterogeneity of mutations that belong to the same class (Harilaou, Plymouth, Santiago, etc.) is associated with the location of each mutation and the loss of G6PD enzyme activity that is caused in each case. In this study, we report the second known case of the G6PD Harilaou variant, which manifested with intrauterine haemolysis.

## Case report

A male neonate was born to a G2P2 mother at 36 weeks of gestation with an emergency caesarean section due to an abnormal foetal nonstress test. According to Fenton preterm growth charts [7], the baby’s values were the following: birth weight [2870g (66th percentile)], length [49 cm (86th percentile)], and head circumference [34.5 cm (93rd percentile)]. During labour, the amniotic fluid was meconium stained. The neonate was severely compromised, experienced respiratory distress and required resuscitation with intubation in the delivery room. The Apgar score was 1’ [2], 5’ [4], and 10’ [5]. After stabilisation, the newborn was transferred to the NICU in critical condition. Meconium aspiration syndrome (MAS) was detected, along with persistent pulmonary hypertension and metabolic and respiratory acidosis (ABG: pH 6.81, pCO2 80.5 mmHg, HCO3- 9 mmol/L, pO2 30.2 mmHg, ABE -24 mmol/L, cLac 85 mg/dl).

Due to severe respiratory insufficiency and an oxygenation index above 25, the newborn was directly subjected to high-frequency oscillatory ventilation (HFOV) and nitric oxide administration. The first settings on the HFOV device were the following: FiO2 100%, PIP 35 cmH2O, PEEP 6 cmH2O, BPM 60/min, IT 0.42 sec. In addition, the head ultrasound showed diffused cerebral oedema. Laboratory tests revealed persistent anaemia and failure to sustain a Ht of more than 22% despite three blood transfusions. The cord blood haemoglobin was 7 g/dl, and the reticulocyte count was 41%—findings that are consistent with haemolytic anaemia. The Coombs test was negative, excluding autoimmunity. There was no hydrops nor jaundice. It is essential to highlight that all previous laboratory exams and nonstress tests were normal, suggesting an uncomplicated pregnancy. A review of all findings showed that intrauterine haemolysis had occurred. The family history of the maternal uncle, who was the first reported G6PD Harilaou case, led to further clinical investigations, which revealed G6PD deficiency in the neonate. The baby’s poor general condition, which was attributed to severe intrauterine hypoxia due to anaemia and MAS, remained. Cardiopulmonary insufficiency developed, and unfortunately, the newborn passed away 39 hours postbirth due to cardiac arrest. Following death, blood samples and skin patches were taken for examination and were forwarded to a specialised centre. PCR and DNA sequencing of the baby’s blood led to the final diagnosis, as the G6PD Harilaou variant was identified (c.738T>G. p.Phe216Leu). The variant was not detected in the elder brother, who was also tested.

## Discussion

The case we demonstrate is the second reported case of a patient with the G6PD Harilaou variant. Remarkably, in our patient the mutation had a more profound clinical appearance. The Harilaou variant can be compared to the Guadalajara variant (Class I, WHO) [8], which manifested with haemolysis, foetal anaemia and jaundice. However, the baby of the published Guadalajara case had a favourable outcome. In our case, G6PD deficiency in the maternal uncle became clinically obvious at the age of three years with the manifestation of transfusion-refractory anaemia. However, he underwent a splenectomy and survived. Today he is about 38 years old and coping with chronic anaemia via periodic transfusions when required. Our patient’s clinical manifestations did not match the usual asymptomatic profile of the G6PD Mediterranean variant. The baby suffered intrauterine haemolysis, developing chronic haemolysis and hypoxia. MAS followed, as hypoxia and acidosis are strong stimulants for meconium passage in utero (a condition known as meconium-stained amniotic fluid (MSAF)), owing to vagal stimulation and anal sphincter relaxation. MSAF complicates almost 10–15% of childbirths, while MAS occurs in 5% of them [9]. MAS is a clinical syndrome defined by respiratory distress in a neonate born with MSAF, a need for oxygen to maintain SaO2 at 92% or more, oxygen requirements that start during the first two hours of life and that last for at least 12 hours, and the absence of congenital cardiac or pulmonary malformations [10]. The MSAF is aspirated from the foetus and fills the airways, leading to severe respiratory distress and pulmonary hypertension. MAS pathophysiology combines pulmonary dysfunction with a systemic inflammatory response. Persistent pulmonary hypertension, though, remains the leading cause of death in MAS, as happened in our case.

**Figure 1:**
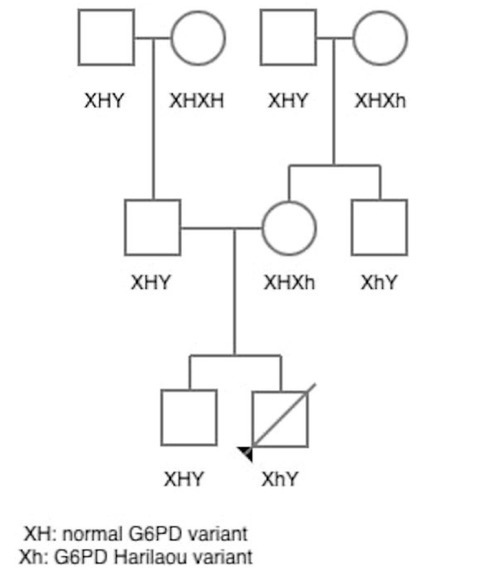
Family pedigree

In the family pedigree (fig. 1), it is obvious that the newborn inherited the G6PD Harilaou variant from his mother, who was found to be a heterozygous carrier [11]. Thus, her sons inherited a normal and a Harilaou X-linked variant, respectively [12]. We conclude that the maternal grandmother was heterozygous for the Harilaou variant.

## Conclusion

G6PD deficiency can be caused by several mutations, leading to a spectrum of mild, moderate or severe clinical presentation. Some of them can lead to life-threatening chronic haemolysis and neonatal death. Although the Mediterranean variant prevails in Greece, clinicians should always stay alert to detect carriers of variants with a more severe phenotype. This case highlights the need for intensified monitoring in women with a family history of this disorder in order to ensure the wellbeing of the foetus.

### Bulleted key points

- This is the second published case of a patient with G6PD deficiency and Harilaou variant.
- G6PD Harilaou is a rare and potentially lethal variant that may cause intrauterine haemolysis.
- G6PD variants are related, with various enzyme activity and severity of haemolysis.
- Women with a family history of G6PD deficiency and haemolysis should be offered intensified foetal monitoring.
